# Protein Coevolution and Isoexpression in Yeast Macromolecular Complexes

**DOI:** 10.1155/2007/58721

**Published:** 2007-01-08

**Authors:** Laurence Ettwiller, Reiner A. Veitia

**Affiliations:** ^1^CNRS UMR 7637, Ecole Supérieure de Physique et de Chimie Industrielles, 10 rue Vauquelin, 75005 Paris, France; ^2^European Molecular Biology Laboratory Heidelberg, Meyerhofstraße 1, 69117 Heidelberg, Germany; ^3^Institut Cochin, 75014 Paris, France; ^4^INSERM U567, 75014 Paris, France; ^5^CNRS UMR 8104, 75014 Paris, France; ^6^Faculté de Médecine René Descartes, Université Paris 5, UM 3, 75014 Paris, France; ^7^UFR de Biologie et Sciences de la Nature, Université Paris 7, 75005 Paris, France

## Abstract

Previous studies in the yeast *Saccharomyces cerevisiae* have shown that genes encoding subunits of macromolecular complexes have similar evolutionary rates (K) and expression levels (E). Besides, it is known that the expression of a gene is a strong predictor of its rate of evolution (i.e., E and K are correlated). Here we show that intracomplex variation of subunit expression correlates with intracomplex variation of their evolutionary rates (using two different measures of dispersion). However, a similar trend was observed for randomized complexes. Therefore, using a mathematical transformation, we created new variables capturing intracomplex variation of both E and K. The values of these new compound variables were smaller for real complexes than for randomized ones. This shows that proteins in complexes tend to have closer expressivities (E) and K's simultaneously than in the randomly grouped genes. We speculate about the possible implications of this finding.

## 1. INTRODUCTION

Many factors are likely to influence the rate of protein evolution in yeast. For instance, essential genes, lethal when deleted, have been reported to evolve more slowly than nonessential ones (Hirsh and Fraser [1], but see also Pál et al. [2]). Another related issue is the negative correlation between the number of protein-protein interactions (connectivity) and evolutionary rate. Accordingly, highly connected proteins would evolve slowly, independently of their essential or dispensable character (Fraser et al. [3, 4]). This has been reassessed several times, but according to Bloom and Adami [5], the negative correlation between connectivity and evolutionary rate would be the result of biases in protein-protein interaction datasets (Bloom and Adami [5]).

It is also known that highly expressed genes evolve at lower rates (Pál et al. [6]). Indeed, the number of transcripts of each gene per cell (called here expressivity: E) is negatively correlated with the rate of protein evolution, estimated from the number of nonsynonymous substitutions per nonsynonymous amino acid site (K). According to Bloom and Adami [5], this is the only correlation that withstands a careful analysis using several datasets. Nevertheless, the forces behind this negative relationship remain largely unknown.

In the case of interacting proteins, one expects them to evolve at similar rates because a change in one protein would necessitate compensatory changes in the others to ensure the persistence of the interactions. Based on that notion, Fraser et al. [3], have compared the distribution of the differences between the K (i.e., DK) of interacting proteins with the distribution of DK for random protein pairs. They were able to show that interacting proteins in yeast have similar evolutionary rates. Therefore, they suggested that coevolution of protein subunits is due to compensatory mutations stemming from their mutual interactions.

From the perspective of expression, there is evidence for similar expressivity (i.e., isoexpression) at the mRNA level of the subunits of several yeast stable complexes (Jansen et al. [7]). This is not surprising and might stem from the stoichiometric balance that the subunits of a complex should respect to avoid fitness defects (Veitia [8]; Papp et al. [9]). Here, we reexplore the link between coevolution and similar expression levels of the subunits of the same protein complexes. We expect to find a significant positive correlation between a measure of dispersion of E and K of proteins involved in complexes. That is, intracomplex variation of E is expected to correlate with intracomplex variation of K. Specifically, we have explored 94 complexes composed of 4 or more subunits, extracted from the MIPS catalogue. We considered manually annotated stable complexes. The K values used were those obtained from comparisons between orthologs of *Saccharomyces cerevisiae* and *Candida albicans* or *Schizosaccharomyces pombe* and were provided by H. Fraser (Fraser et al. [4]). The levels of expression were those estimated by Holstege et al. [10]. Moreover, as there may be biases in the estimation of the absolute gene expression levels, we also used the codon adaptation index (CAI), a measure of synonymous codon usage bias, as a proxy of E. Indeed, CAI is strongly correlated with E, as synonymous codon usage is supposed to be coadapted with isoacceptor tRNA pools to enhance the efficiency of protein synthesis (Sharp and Li [11]).

## 2. RESULTS AND DISCUSSION

In accordance with previous studies (Bloom and Adami [5] and references therein), we found a highly significant correlation between the mean E of the complexes and their mean K (Pearson's *R* = −0.45, *p* < 10^−6^, *n* = 94, using K_*Candida-Saccharomyces*_). Interestingly, the correlation between mean(logE) versus mean(logK) was even stronger (*R* = −0.77, *p* < 10^−20^, *n* = 94). Similar results were obtained for the correlation between CAI and K (data not shown) suggesting that E and CAI can be interchangeably used in this type of study.

In order to assess the link between intracomplex variation of E (or CAI) and intracomplex variation of K, we used two measures of dispersion: the traditional standard deviation (SD) and the median normalized difference *DXij = |Xi−Xj|*/(*Xi+Xj*), where *X* is any property (K, E, or CAI) of subunits *i* and *j* of the complex (Jansen et al. [7]). Note that *DX* is calculated for all combinations of subunits whether they interact directly or not. Our analysis is different in its essence from that of Fraser et al. [3], as they pooled all binary interactions (of all complexes studied). Here, we have considered the complexes as individual entities.

In our analysis, we noted that the mean E and mean K for the complexes correlated strongly and significantly with their corresponding standard deviations. In order to break or diminish these correlations, a logarithmic transformation was applied to the values of both E and K for each gene before computing the standard deviations for each complex (Keene [12]). This transformation was further justified by the fact that the correlation between mean (logK) and mean (logE) for the complexes was stronger than for the nontransformed data. In short, we used SD(logE) and SD(logK) for each complex as measures of dispersion. Lastly, for comparison with random expectation, we created a sample of groups of genes randomly chosen from those involved in complexes. The sample of random “complexes” was similar to the real ones in number and size. We used a median test to estimate the *P* values for the differences between the real and random samples.

**Figure 1:**
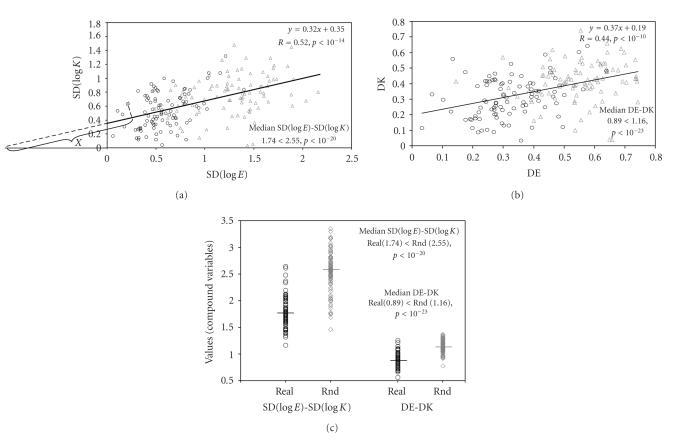
*The expression levels and the evolutionary rates of the subunits of macromolecular complexes are closer than expected by random.* (a) Regression analysis of median SD(logE) and median SD(logK). (b) Regression analysis of median DE and median DK. The K values were those obtained from comparisons between orthologs of *S cerevisiae* and *C albicans*. However, very similar results were obtained for K values drawn from comparisons between orthologs of *S cerevisiae* and *S pombe*. Black circles represent 94 real complexes and gray triangles represent a sample of 94 groups containing randomly chosen genes treated in the same way as complexes. A regression line, common to real and random complexes, appears in black. Note that the points representing the real complexes concentrate in the lower-left quadrant. To test this statistically, we defined a new composite variable as the distance on the regression line between its intersection with the x-axis and the orthogonal projection of the data points on the regression line (e.g., the distance “*X*” in Figure 1(a)). (c) Distribution of the values of the compound variables SD(logE)-SD(logK) and DE-DK for real and random (Rnd) complexes. The medians of the composite variables (represented by horizontal lines) for real complexes and random groups were statistically different.

We found that the median SD(logK) of real complexes was smaller than that of the randomized dataset (Table 1). This result confirms by an independent method a previous claim that proteins in complexes tend to have closer evolutionary rates (Fraser et al. [3]). As expected, we also found similar results for the expressivities (E) of the ORFs encoding subunits of stable complexes (Table 1). Furthermore, we detected a positive and significant correlation between SD(logE) and SD(logK) for the real complexes (i.e., *R* = 0.46, *p* < 10^−6^, *n* = 94, using K_*Candida-Saccharomyces*_). A significant correlation between SD(logE) and SD(logK) also appeared in the regression involving the sample of randomly grouped genes (i.e., *R* = 0.31, *p* < 0.003, *n* = 92, using K_*Candida-Saccharomyces*_). Both correlation coefficients were statistically similar (*p* = 0.23). The correlation in the random gene groups is likely to be explained by the strong correlation between logK and logE which also holds, of course, for real complexes. Nevertheless, Figure 1 shows that the cloud of points representing the real complexes seemed to have smaller dispersions for both E and K values at the same time (i.e., the points tend to concentrate in the lower left “quadrant”). To test this statistically, we created a new variable capturing the information of both SD(logK) and SD(logE) simultaneously. The new compound variable (i.e., SD(logK)-SD(logE)) was defined as the distance on the regression line between its intersection with the x-axis and the orthogonal projection of the data points on the regression line. The statistical comparison of the medians of the composite SD(logK)-SD(logE) variable showed that real complexes had smaller values than random gene groups (Table 2). This clearly shows that proteins in complexes tend to have closer expressivities and K's simultaneously than those from randomly grouped genes. Similar findings were obtained using the second measure of dispersion, namely, the median normalized differences DE and DK (Table 2). Since CAI correlates very strongly with E, a similar analysis using CAI as a proxy of E recapitulated the results obtained for the latter (Table 2). We have also excluded the potential trivial effect of the size of the complexes (i.e., small complexes can have either very high or very low variances) as there is no correlation between any of the composite variables and the size of the complexes analyzed.

Genes in yeast have been divided into essential or dispensable according to the effect of their homozygous deletions in certain experimental conditions (i.e., resp., lethal or not, Giaever et al. [13]). Moreover, essential genes also tend to be highly connected and to be central in the protein network (Wuchty and Almaas [14]). Thus, we have assessed the impact of gene essentiality on our results. Specifically, we concentrated on either essential or dispensable genes for each complex, when the number of relevant subunits was > 4. We failed to detect differences between the median K or the median E for both sets (i.e., essential versus dispensable). This suggests that essentiality has a limited impact on the rate of evolution of proteins involved in the macromolecular complexes that we have analyzed. This lends credence to previous claims (Pál et al. [2]). When analyzing the measures of dispersion, essential and dispensable subunits followed the same general trend documented above for the whole complexes and had statistically similar values of the composite variables SD(logK)-SD(logE) and DE-DK (data not shown).

On general grounds, several mechanisms might explain the (nonlinear) relationship between E and K. For instance, on the one hand, transcription rate correlates with the frequency of spontaneous mutation in yeast (Morey et al. [15]) but on the other hand, transcription-blocking mutations are usually repaired faster than lesions in the nontranscribed strand or in the overall genome (Svejstrup [16]). In the same way that there has been selection to shape the synonymous codon usage in yeast, there is evidence for selection at nonsynonymous sites to enhance the rate and accuracy of translation. For instance, the tightness of the correlation between tRNA gene numbers and amino acid usage increases as a function of the expression levels. Thus, translational selection could provide a further explanation for the negative correlation between K and E (Akashi [17]). Our results show that in *Saccharomyces cerevisiae*, the link between protein coevolution and isoexpression deserves credence. We detect not only a correlation between the average E (or CAI) and K but also between their dispersions. Moreover, the combined measures of dispersion of both E and K (i.e., composite variables) are smaller than expected by chance. In other words, the tighter the regulation of the expression (as judged by the RNA levels) of the subunits of a complex is, the closer the evolutionary rates of these components will be. It is tempting to propose that tuning the expression levels of the various subunits of the complexes, to avoid stoichiometric imbalances, shapes their evolution. This might be achieved by selection of specific patterns of both synonymous and nonsynonymous codon usage to ensure similar expression levels, yet respecting the residues involved in physical interactions. However, our results do not exclude the possibility that a third covariate might dictate E and K. Indeed, more mechanistic studies (i.e., not only genomic surveys) are required to work out the causal relationships.

## Figures and Tables

**Table 1:** *The median dispersions of K and E or CAI are smaller for complexes than for random gene groups.* The K Cal and K Spo are those obtained from comparisons between orthologs of *S cerevisiae* and *C albicans* (Cal) or *Sch Pombe* (Spo), respectively, *P* values from a median test.

Medians	SD(logE)	SD(logCAI)	SD(logK) Cal	SD(logK) Spo	DE	D CAI	DK Cal	DK Spo

Observed	0.53	0.18	0.51	0.46	0.30	0.11	0.30	0.27
Random	1.33	0.55	0.78	0.72	0.56	0.27	0.42	0.39
*P* (two-tailed)	1.0E-19	1.0E-18	1.0E-06	1.0E-06	1.0E-20	1.0E-20	1.0E-07	1.0E-07

**Table 2:** *The median values of the different composite variables are smaller for complexes than for random gene groups.* The K Cal and K Spo are those obtained from comparisons between orthologs of *S cerevisiae* and *C albicans* (Cal) or *Sch pombe* (Spo), respectively, *P* values from a median test.

Medians	SD(logE)-SD(logK) Cal	SD(logE)-SD(logK) Spo	SD(logCAI)-SD(logK) Cal	SD(log) CAI-SD(logK) Sp**o**	DE-DK Cal	DE-DK Spo	D CAI-DK Cal	D CAI-DK Spo

Observed	1.74	1.64	0.86	0.79	0.89	0.79	0.55	0.49
Random	2.55	2.43	1.33	1.25	1.16	1.06	0.75	0.68
*P* (two-tailed)	1.0E-20	1.0E-20	1.0E-14	1.0E-16	1.0E-23	1.0E-19	1.0E-17	1.0E-16
